# Effects of an Iodine-Containing Prenatal Multiple Micronutrient on Maternal and Infant Iodine Status and Thyroid Function: A Randomized Trial in The Gambia

**DOI:** 10.1089/thy.2019.0789

**Published:** 2020-09-08

**Authors:** Kamilla G. Eriksen, Maria Andersson, Sandra Hunziker, Michael B. Zimmermann, Sophie E. Moore

**Affiliations:** ^1^ Department of Nutrition, Exercise and Sports, University of Copenhagen, Copenhagen, Denmark.; ^2^ Division of Gastroenterology and Nutrition, Children's Research Centre, University Children's Hospital Zurich, Zurich, Switzerland.; ^3^ Human Nutrition Laboratory, Institute of Food, Nutrition, and Health, ETH Zurich, Zurich, Switzerland.; ^4^ Department of Women and Children's Health, King's College London, London, United Kingdom.

**Keywords:** iodine, thyroid, pregnancy, lactation, infancy

## Abstract

***Background:*** Iodine supplementation is recommended to pregnant women in iodine-deficient populations, but the impact in moderate iodine deficiency is uncertain. We assessed the effect of an iodine-containing prenatal multiple micronutrient (MMN) supplement in a rural Gambian population at risk of moderate iodine deficiency.

***Materials and Methods:*** This study uses data and samples collected as a part of the randomized controlled trial Early Nutrition and Immune Development (ENID; ISRCTN49285450) conducted in Keneba, The Gambia. Pregnant women (<20 weeks gestation) were randomized to either a daily supplement of MMNs containing 300 μg of iodine or an iron and folic acid (FeFol) supplement. Randomization was double blinded (participants and investigators). The coprimary outcomes were maternal urinary iodine concentration (UIC) and serum thyroglobulin (Tg), assessed at baseline and at 30 weeks' gestation. Secondary outcomes were maternal serum thyrotropin (TSH), total triiodothyronine (TT3), total thyroxine (TT4) (assessed at baseline and at 30 weeks' gestation), breast milk iodine concentration (BMIC) (assessed at 8, 12, and 24 weeks postpartum), infant serum Tg (assessed at birth [cord], 12, and 24 weeks postpartum), and serum TSH (assessed at birth [cord]). The effect of supplementation was evaluated using mixed effects models.

***Results:*** A total of 875 pregnant women were enrolled between April 2010 and February 2015. In this secondary analysis, we included women from the MMN (*n* = 219) and FeFol (*n* = 219) arm of the ENID trial. At baseline, median (interquartile range or IQR) maternal UIC and Tg was 51 μg/L (33–82) and 22 μg/L (12–39), respectively, indicating moderate iodine deficiency. Maternal MMN supplement increased maternal UIC (*p* < 0.001), decreased maternal Tg (*p* < 0.001), and cord blood Tg (*p* < 0.001) compared with FeFol. Maternal thyroid function tests (TSH, TT3, TT4, and TT3/TT4 ratio) and BMIC did not differ according to maternal supplement group over the course of the study. Median (IQR) BMIC, maternal UIC, and infant Tg in the MMN group were 51 μg/L (35–72), 39 μg/L (25–64), and 87 μg/L (59–127), respectively, at 12 weeks postpartum, and did not differ between supplement groups.

***Conclusions:*** Supplementing moderately iodine-deficient women during pregnancy improved maternal iodine status and reduced Tg concentration. However, the effects were not attained postpartum and maternal and infant iodine nutrition remained inadequate during the first six months after birth. Consideration should be given to ensuring adequate maternal status through pregnancy and lactation in populations with moderate deficiency.

## Introduction

Iodine is an essential substrate for the production of thyroid hormone and adequate iodine nutrition is especially important during the first 1000 days of life, when the risk of deficiency for the fetus and infant is high. Infants are particularly sensitive to iodine deficiency because they have the highest production of thyroid hormones per kilogram body weight, and are born with minimal thyroidal iodine stores (1). Exclusively breastfed infants rely on iodine from breast milk alone to cover their high rates of thyroid hormone production (2).

Severe iodine deficiency during pregnancy may result in maternal and fetal hypothyroidism, increased risk for infant and perinatal mortality, pregnancy loss, maternal and fetal goiter, and growth retardation (2). Thyroid hormone is critical for fetal and infant neurodevelopment, and severe iodine deficiency during pregnancy is associated with neurologic deficits and cretinism in children (3,4). Mild-to-moderate iodine deficiency may affect maternal and fetal thyroid function (5), but the impact on neurodevelopmental outcomes in offspring remains uncertain (4,6,7). Iodine deficiency during infancy may also result in altered thyroid function and impaired brain development, but data are limited (8).

Salt iodization is the primary intervention strategy to prevent iodine deficiency in the general population (9). However, poor coverage of iodized salt or use of noniodized alternatives may increase the risk for mild-to-moderate iodine deficiency during the first 1000 days (from conception to the child's second birthday) when dietary iodine requirements are high (10). In The Gambia, the coverage of adequately iodized salt is poor, and a recent nationally representative cross-sectional survey found rural pregnant women to be iodine deficient (11).

Iodine supplementation of pregnant and lactating women is recommended in iodine-deficient populations wherein salt iodization is insufficient (12). Iodine supplementation during pregnancy in mildly iodine-deficient women improves maternal iodine status, thyroid volume, and thyroid indices (5,7). However, studies conducted in moderately iodine-deficient populations are small and did not follow women and infants after delivery (4,5,13,14). A growing body of evidence demonstrates the beneficial effects of prenatal multiple micronutrient (MMN) supplements, particularly in women entering pregnancy with a poor nutritional status (15–17). Iodine is commonly added to MMN supplements, but to our knowledge, the specific impact of iodine delivered in an MMN supplement on iodine status and thyroid function has not been evaluated in moderately iodine-deficient pregnant and lactating women and infants.

The aim of this study was to investigate the effect of an iodine-containing MMN supplement given during pregnancy (providing 300 μg of iodine) in a rural Gambian population exposed to moderate iodine deficiency. We hypothesized that iodine supplementation would improve maternal iodine status and thyroid function in pregnant women and that the impact would last postpartum and improve breast milk iodine concentration (BMIC) and infant iodine status.

## Materials and Methods

The current analysis used data and samples collected as part of the Early Nutrition and Immune Development (ENID) trial (ISRCTN49285450), a randomized trial conducted in The Gambia between April 2010 and February 2015.

The objective of the main trial was to assess the effect of combined prenatal and infant nutritional supplementation on infant immune development (18). Full details of the ENID trial have been described in detail in the published trial protocol (18). In brief, pregnant women (aged 18–45 years) from the rural West Kiang region of The Gambia were enrolled. Exclusion criteria were gestational age at enrolment ≥20 weeks, multiple pregnancy, severe anemia (hemoglobin <7 g/dL), or confirmed as HIV positive. When scheduled for prenatal care, pregnant women were randomized to four intervention groups of prenatal dietary supplements: (a) MMNs, (b) iron and folic acid (FeFol = standard care), (c) protein energy (PE), and (d) PE + MMN. Supplementation continued until delivery. In this secondary analysis, we included participants from the two prenatal tablet arms (a) MMN and (b) FeFol). This decision was made on the basis of evidence of differential adherence between the tablet and the lipid-based nutritional supplement (LNS) groups, with significantly lower adherence to supplementation in the LNS groups (19). In ENID, infants were further supplemented with daily LNS or LNS + micronutrients after six months of age. However, the infant intervention arms will not be described here as the present analyses stops at six months after delivery.

The protocol of the original ENID trial was approved by the joint Gambia Government/Medical Research Council (MRC) Unit, The Gambia Ethics Committee (Project No. SCC1126v2). Written informed consent was obtained from all women before enrolment into the trial. The trial observed good clinical practice standards and the current version of the Helsinki Declaration.

### Randomization

The women included in this subanalysis of the ENID trial were randomized to one of the two following intervention arms:

1. MMNs, a combination of 15 micronutrients, specifically designed for use during pregnancy as formulated by the World Health Organization (WHO), United Nations University, and United Nations Children's Fund (20), and containing twice the recommended daily allowance for all contained micronutrients, with the exception of FeFol that was set at Gambian Government Guidelines (Supplementary Table S1). The MMN supplement contained 300 μg iodine as potassium iodide.
2. FeFol, representing the usual standard of care during pregnancy as per Gambian Government Guidelines (iron 60 mg/day, folic acid 400 μ/day), with no iodine.

The MMN and FeFol supplements were formulated as tablets and manufactured by Scanpharm, Birkerød, Denmark. The iodine content of the MMN supplement was not verified by independent laboratory testing.

Randomization into the trial was performed in blocks of 8, using an automated system, with the 8 groups reflecting the 8 combinations of prenatal and infancy supplements. The prenatal arm of the full ENID trial was partly open, as it was not possible to blind the field assistants or the women to the supplement type (tablet vs. LNS). However, for the purpose of this analysis, wherein only the two prenatal tablet arms are considered, the trial can be considered as double blinded as the tablets were identical.

### Procedures

Clinical visits were performed at baseline, 20 and 30 weeks' gestation, at birth, and 1, 8, 12, 24, and 52 weeks postpartum. Women received an MMN tablet containing 300 μg iodine or a FeFol tablet without iodine, taken once daily at baseline (<20 weeks' gestation) until delivery. Field assistants provided the prenatal supplements on a weekly basis. Compliance was assessed through a count of remaining tablets at the end of each week, and an average weekly compliance was calculated to assess study mean compliance for each group. We assessed side effects by use of a questionnaire at these weekly visits. Serious adverse events were defined as death or hospital admission of either mother or infant for a cause other than delivery. At baseline, a structured questionnaire was administered to collect data on general subject characteristics, race/ethnicity, and educational level.

At baseline, participants' height and weight were measured and gestational age was determined by ultrasound. Body mass index (BMI) was calculated by bodyweight (kg) divided by height (m) squared. A venous blood sample collected from the women after an overnight fast at baseline (<20 weeks' gestation) and 30 weeks' gestation, for measurement of maternal thyroid function, was used in this analysis. The blood samples were immediately put on ice, and then centrifuged, aliquoted, and stored at −80°C. An 24-hour urine sample was collected for measurement of maternal urinary iodine concentration (UIC) at baseline, at 30 weeks' gestation, and 12 weeks postpartum. A field worker visited the women's home every four hours during the day to collect the urine samples (which were stored on ice), and transported the samples to the MRC Keneba field station where they were refrigerated. At the end of 24 hours, the urine samples from each individual woman were pooled, aliquoted, and stored at −20°C.

Immediately after delivery, the placenta was passed to an attending field assistant and a blood sample collected from the umbilical vein. If the woman delivered at home, the sample was put on ice and transported to the MRC Keneba field station. On arrival in the laboratory, samples were centrifuged, aliquoted, and stored at −80°C until processing. Infant birth weight and length were obtained within 72 hours after delivery, by using electronic scales (Seca 336) and length boards (Seca 417), which were precise to 10 g and 1 mm, respectively. Head circumference was also measured at birth, using a circumference measuring tape (Seca 201). Low birth weight is defined as weight at birth of <2500 g irrespective of gestational age (21), preterm birth as gestational age at birth of <37 completed weeks, stunting as height-for-age >2 standard deviations (SDs) below the WHO Child Growth Standards median (22), and wasting as weight-for-length < −2 SDs.

At 8, 12, and 24 weeks postpartum, the women provided a 5 mL breast milk sample from each breast. The samples from right and left breasts were pooled for analysis. The breast milk sample was not collected during a feed or standardized according to the infant's last feed, and was, therefore, a mixture of hind- and foremilk. The breast milk sample was manually expressed between ∼9 and 11 a.m. at the MRC Keneba field station, and immediately put on ice, and stored at −80°C. The majority of the women were fasting when the milk sample was collected, as breakfast (provided at the MRC clinic) was served after the last sample collection. Throughout the trial, participants were asked weekly about breastfeeding practices and introduction of complementary foods. Exclusive breastfeeding (EBF) was defined according to the WHO definition: no other foods or liquids consumed than breast milk with the exception of medicines, or essential vitamins or minerals.

A venous blood sample from each participating infant at 12 and 24 weeks postpartum was used in this analysis. Samples of infant blood were collected by venipuncture, and immediately put on ice before being centrifuged, aliquoted, and stored at −80°C.

The coprimary outcomes in this analysis were maternal UIC and serum thyroglobulin (Tg) concentration (baseline and 30 weeks' gestation). Secondary outcomes were maternal UIC at 12 weeks postpartum, serum thyrotropin (TSH), total triiodothyronine (TT3), total thyroxine (TT4), TT3/TT4 ratio (baseline and 30 weeks' gestation), thyroglobulin antibodies (TgAbs) (baseline), and BMIC (weeks 8, 12, and 24 postpartum) as well as infant serum Tg concentration (birth [cord], 12, and 24 weeks postpartum) and serum TSH in cord blood.

### Sample analysis

UIC was measured using inductively coupled plasma mass spectrometry (ICP-MS) (23), at the Human Nutrition Laboratory of Eidgenössische Technische Hochschule (ETH) Zurich (Zurich, Switzerland). WHO criterion based on the median UIC was used to classify adequate iodine intake for pregnant women (≥150 μg/L) (9).

BMIC was measured by ICP-MS at MRC Elsie Widdowson Laboratory (Cambridge, UK). Breast milk samples were first diluted (1:50) with a solution of ultragrade tetramethylammonium hydroxide (TMAH) containing tellurium as internal standard (0.5% TMAH, 20 μg/L tellurium). The samples were then analyzed by ICP-MS along with external matrix-matched calibration standards (commercially sourced pooled breast milk; Sera Laboratories International, Ltd.). Serum and whole blood (RECIPE Chemicals+, Instruments GmbH and Sero AS) were used as quality controls.

Tg was measured in maternal and infant serum using a sandwich serum-Tg enzyme-linked immunosorbent assay (ELISA) (24), at the Human Nutrition Laboratory of ETH Zurich. Liquicheck™ Tumor Marker Control (Bio-Rad Laboratories AG, Cressier, Switzerland; LOT. 19990 and LOT. 19970) was used as the standard. Elevated Tg concentrations during pregnancy, indicating iodine deficiency, is defined as Tg >43.5 μg/L (24,25).

Serum TSH, TT3, TT4, and cord blood TSH were measured by immunoassay (IMMULITE; Siemens Healthcare Diagnostics, UK) at the Human Nutrition Laboratory of ETH Zurich using analyte-specific kits and controls. For TSH during pregnancy, we used trimester-specific reference ranges: 0.1–2.5 mIU/L for the first trimester, 0.2–3.0 mIU/L for the second trimester, and 0.3–3.0 mIU/L for the third trimester (26). For TT4 until gestational week 6, we used the reference range of 58–161 nmol/L; from week 7, we increased the upper reference range by 5% per week until week 15; from week 16 until delivery, we multiplied the nonpregnancy reference range by 1.5 and used the resulting range of 87.0–241.5 nmol/L as a reference (26). For TT3, we used the manufacturer's reference ranges of 1.3–2.6 nmol/L.

Subclinical hypothyroidism was defined as a high TSH and a normal TT4, overt hypothyroidism was defined as a high TSH and a low TT4, overt hyperthyroidism was defined as a low TSH and a high TT4, subclinical hyperthyroidism was defined as low TSH and normal TT4, and isolated hypothyroxinemia was defined as a normal TSH and a low TT4.

Maternal TgAb concentrations were analyzed in baseline samples using a serum ELISA (TgAb ELISA, version 2; RSR, Cardiff, UK). The manufacturer cutoff for TgAb positivity is ≥65 U/mL.

The interassay variability for all analyses are reported in Supplementary Data.

### Statistical analysis

Full details of the power calculations applied are provided in the published trial protocol (18). A *post hoc* power calculation was made based on maternal Tg concentrations from a recent trial of mildly iodine-deficient pregnant women (7), using an SD of 7.5 μg/L. In this study, a total of 175 samples in each supplement arm give 96% power to detect a difference of 3 μg/L or more between the MMN and FeFol arm.

The data were analyzed using STATA version 15 and R (27). Descriptive statistics were applied for all variables. Outliers were identified and removed after visual inspection of box plots stratified by group and time point. Values in the text and tables are presented as mean (SD) for normally distributed data, median (interquartile range or IQR) for non-normal data, and number (%) for prevalence. Baseline characteristics of the study population according to maternal supplement groups were assessed by unpaired *t*-tests for parametric data, Mann–Whitney *U*-test for nonparametric data, and Fisher's exact test for categorical dependent variables.

We assessed the intervention effect by fitting individual linear mixed effects models to continuous dependent variables using maximum likelihood procedure for the estimation of variance components. For each variable, an individual mixed effects model was derived with time (two visits for maternal UIC, Tg, TSH, TT3, TT4, infant Tg, and three visits for BMIC), coded as a categorical variable, and maternal supplementation group (MMN or FeFol) as fixed effects. An interaction between time and supplementation was included in the mixed effects models. Between-individual variation was modeled using random effects. For categorical dependent variables, mixed effects logistic regression models were used, using R. For some of the categorical variable analyses, prevalence of thyroid disorders was nonexisting at one or several time points and, therefore, time was not included in these mixed effects logistic regression models.

The residuals were tested for normality and homogeneity of variance using residual plots, and non-normally distributed data were log-transformed and then reanalysed. Outliers were defined as data with residuals >3 SDs from the mean in the linear mixed effects models and were excluded from the models (UIC *n* = 5 data points removed, maternal Tg *n* = 3, maternal TSH *n* = 13, TT3 *n* = 4, TT4 *n* = 5, BMIC *n* = 4, infant Tg *n* = 5). These outliers were not excluded from the mixed effects logistic regression models.

Interactions between time and supplementation for the linear mixed effects model on BMIC were assessed by likelihood-ratio tests between two nested linear mixed effects models, one model with and the other without the interaction terms. The overall supplementation effect for BMIC independent of time was assessed by a likelihood-ratio test comparing two nested mixed effects models, one with maternal supplement group (and its time interaction) and the other without maternal supplement group (i.e., with fixed effects for time only). The likelihood-ratio test tests whether the model including maternal supplement group as a predictor gave a significantly better fit to the data than that without.

The Mann–Whitney *U*-test was used to assess group differences in Tg and TSH at birth (cord blood), and maternal UIC at 12 weeks postpartum. Estimated daily maternal iodine intake was calculated using daily iodine excretion at baseline (using UIC and measured urine volume of the 24-hour urine excretion) and assuming an average iodine bioavailability of 90% (28).

Statistical significance was set at *p* < 0.05.

## Results

**FIG. 1.**
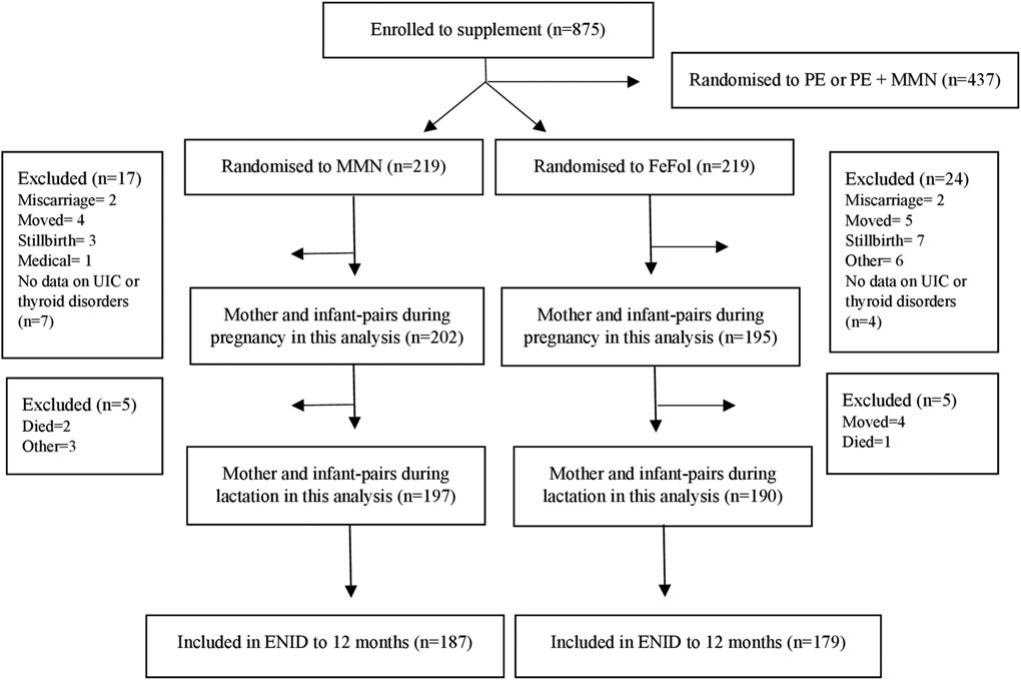
Trial profile for the ENID MMN and FeFol supplement groups and data included in this analysis. ENID, Early Nutrition and Immune Development; FeFol, iron and folic acid; MMN, multiple micronutrient; PE, protein energy; UIC, urinary iodine concentration.

A total of 2798 women consented to the ENID study and 875 pregnant women were eligible. In this study, we only included pregnant women randomly assigned from the MMN (*n* = 219) and FeFol arms (*n* = 219). For the analyses conducted during pregnancy, 397 mother–infant pairs were included and 387 mother–infant pairs for the analyses during lactation (Fig. 1).

At baseline, mean age of the participating women was 29.5 years (SD 6.7) and the mean gestational age was 13.7 weeks (3.4) (Table 1). The mean BMI at baseline was 21.1 kg/m^2^ (3.5), and 20% (88 of 437) of the women were underweight (BMI <18.5 kg/m^2^) and 10% (45 of 437) were overweight (BMI ≥25 kg/m^2^). The majority (77%, 329 of 430) of the participating women had received no formal Arabic or English schooling. The study population had a mean parity of 4.1 (2.7). There were no differences in baseline characteristics between women from the two supplement groups. A small, but significant (1.79 cm, *p* = 0.04), difference was observed in height between women who were initially randomized to supplementation, and those who were lost to follow-up, but no other differences in baseline characteristics were observed (data not presented). Compliance rates for women receiving the MMN and FeFol were 93.1% and 95.7%, respectively (19).

**Table 1.** Characteristics of Women at Baseline and Infants at Birth

	N	MMN	FeFol
Maternal age (years)	438	29.1 (6.7)	29.9 (6.7)
Maternal weight (kg)	437	55.4 (9.8)	55.0 (9.0)
Maternal height (cm)	438	162.1 (5.7)	161.6 (6.2)
Maternal BMI (kg/m^2^)	437	21.1 (3.8)	21.0 (3.2)
Gestational age at baseline (weeks)	436	13.7 (3.4)	13.8 (3.4)
Parity	431		
Primiparous		27 (13%)	22 (10%)
Multiparous (≥1 previous pregnancy)		189 (88%)	193 (90%)
Maternal education^a^	430		
No education		159 (73%)	170 (80%)
Low (1–7 years)		28 (13%)	25 (12%)
Medium (8–14 years)		31 (14%)	17 (8%)
Still birth	438	3 (1.4%)	7 (3.2%)
Gestational age at birth	389	40.3 (1.6)	40.1 (1.7)
Gestational age at birth categories	389		
<37 weeks		7 (4%)	6 (3%)
37–40 weeks		70 (36%)	82 (43%)
>40 weeks		120 (61%)	104 (54%)
Infant birth weight	328	3.010 (0.4)	2.992 (0.4)
Birth weight categories			
Low birth weight (<2.5 kg)		15 (9%)	16 (10%)
Normal birth weight (2.5–3.9 kg)		155 (91%)	140 (89%)
High birth weight (≥4.0 kg)		1 (1%)	1 (1%)
Infant birth length (cm)	340	49.5 (2.0)	49.6 (1.8)
WAZ at birth	328	−0.62 (0.9)	−0.65 (0.9)
LAZ at birth	340	−0.10 (1.05)	−0.08 (1.0)
WLZ at birth	320	−0.90 (1.3)	−1.02 (1.2)
Infant head circumference at birth	339	33.2 (1.4)	33.4 (1.4)

Data are *n* (%) or mean (SD).

^a^ Maternal education was defined as completed years of either English or Arabic schooling.

BMI, body mass index; FeFol, iron and folic acid; LAZ, length-for-age z-score; MMN, multiple micronutrient; SD, standard deviation; WAZ, weight-for-age z-score; WLZ, weight-for-length z-score.

Infants were born with a mean birth weight of 3002 g (0.4) and 9.5% (31 of 328) were born with a low birth weight (<2500 g) (Table 1). Infant mean weight-for-age z-score, length-for-age z-score (LAZ), and weight-for-length z-score (WLZ) at birth were −0.64 (0.9), −0.09 (1.0), and −0.96 (1.3) with no difference according to maternal supplement group (Table 1). Mean infant head circumference was 33.3 cm (1.4) at birth. The majority of infants (93%, 359 of 385) were exclusively breastfed to 3 months of age and 31% (121 of 385) to 6 months of age. The mean age of discontinuation of EBF was 5.2 (1.3) months. Age of discontinuation of EBF did not differ between maternal supplement groups (data not presented). Infants were growth faltering, with 23% (81 of 347) stunting (LAZ < −2) and 14% (50 of 347) wasting (WLZ < −2) at 2 years of age.

Maternal median UIC at baseline was 51 μg/L (IQR 33–82), and the estimated median iodine intake was 71 μg/day (44–104), indicating moderate iodine deficiency. Maternal MMN supplementation during pregnancy significantly improved maternal UIC compared with FeFol (*p* < 0.001; Table 2). Maternal median UIC at 12 weeks postpartum was 34 μg/L (22–52) and 39 μg/L (25–64) for the FeFol and MMN groups, respectively (*p* = 0.08). Between 30 weeks' gestation and 12 weeks postpartum, maternal UIC decreased in both supplement groups (*p* < 0.001 for both groups).

**Table 2.** Maternal Iodine Status and Thyroid Function and Disorders During Pregnancy According to Maternal Supplement Group

	N	Baseline	N	30 weeks' gestation	p^*^
UIC (μg/L)					
MMN	171	56 (29–89)	159	90 (45–177)	
FeFol	167	48 (35–80)	156	41 (28–74)	<0.001
Tg (μg/L)					
MMN	186	20.8 (11.3–41.6)	191	16.8 (8.6–32.8)	
FeFol	180	21.8 (12.6–38.2)	184	24.4 (13.1–41.2)	<0.001
TSH (mIU/L)					
MMN	170	0.7 (0.3–1.2)	182	1.1 (0.7–1.6)	
FeFol	162	0.7 (0.4–1.1)	182	1.2 (0.8–1.6)	0.3
TT3 (nmol/L)					
MMN	153	2.5 (0.6)	166	3.1 (0.6)	
FeFol	147	2.5 (0.7)	169	3.1 (0.6)	0.9
TT4 (nmol/L)					
MMN	156	142.9 (35.6)	171	149.2 (24.9)	
FeFol	151	138.0 (42.0)	172	146.0 (26.4)	0.5
TT3/TT4 ratio					
MMN	152	0.018 (0.004)	166	0.021 (0.005)	
FeFol	147	0.019 (0.005)	169	0.021 (0.005)	0.4
Elevated Tg					
MMN	186	23.7% (44)	191	13.1% (25)	
FeFol	180	20.6% (37)	184	22.3% (41)	<0.001
Positive TgAb					
MMN	135	2.2% (3)		—	
FeFol	136	4.4% (6)		—	—
Subclinical hypothyroidism					
MMN	153	0.0 (0)	170	2.9% (5)	
FeFol	145	2.1% (3)	172	2.9% (5)	0.8^**^
Overt hypothyroidism					
MMN	153	0.0 (0)	170	0.0 (0)	
FeFol	145	0.0 (0)	172	0.0 (0)	—
Subclinical hyperthyroidism					
MMN	153	12.4% (19)	170	5.9% (10)	
FeFol	145	9.0% (13)	172	2.3% (4)	0.6
Overt hyperthyroidism					
MMN	153	3.4% (5)	170	0.0 (0)	
FeFol	145	2.1% (3)	172	0.0 (0)	0.5^**^
Isolated hypothyroxinemia					
MMN	153	1.3% (2)	170	0.0 (0)	
FeFol	145	2.8% (4)	172	0.0 (0)	0.4^**^

Data are median (IQR) (non-normally distributed data), means (SD), or % (*n*) derived from raw data. Non-normally distributed data were log-transformed before analysis. Continuous dependent variables were analyzed using linear mixed effects models and categorical dependent variables were analyzed using mixed effects logistic regression models. Subclinical hypothyroidism is defined as high TSH and normal TT4 (relative to gestational age specific cutoffs), overt hypothyroidism is defined as high TSH and low TT4, subclinical hyperthyroidism is defined as low TSH and normal TT4, overt hyperthyroidism is defined as low TSH and high TT4, and isolated hypothyroxinemia is defined as normal TSH and low TT4.

^*^ The *p*-value tests time by supplement interaction.

^**^ This *p*-value is derived without time included in the mixed effects model.

IQR, interquartile range; Tg, thyroglobulin; TgAbs, thyroglobulin antibodies; TSH, thyrotropin; TT3, total triiodothyronine; TT4, total thyroxine; UIC, urinary iodine concentration.

**FIG. 2.**
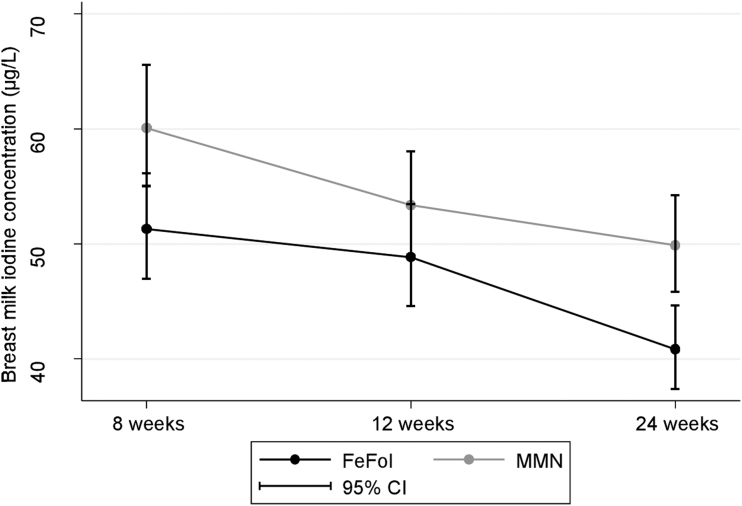
Breast milk iodine concentration (μg/L) (geometric means) according to supplement group at 8, 12, and 24 weeks postpartum. CI, confidence interval.

Median BMIC at 8 weeks postpartum was 54 μg/L (37–79), with 57 μg/L (41–83) and 51 μg/L (35–74) for the MMN and FeFol groups, respectively (Table 3). There were no difference in BMIC between supplement groups over the course of the study (*p* = 0.3; Table 3); however, there was a significant difference in BMIC independent of time (*p* = 0.006), with a higher BMIC in the MMN group (Fig. 2).

**Table 3.** Breast Milk Iodine Concentration and Infant Thyroglobulin Concentration According to Maternal Supplement Group

	N	8 Weeks postpartum	N	12 Weeks postpartum	N	24 Weeks postpartum	p^*^
Breast milk							
BMIC (μg/L)							
MMN	160	57 (41–83)	175	51 (35–72)	175	51 (32–74)	
FeFol	154	51 (35–74)	153	44 (33–73)	157	39 (30–57)	0.3
Infants							
Tg (μg/L)							
MMN		—	163	87 (59–127)	152	67 (42–95)	
FeFol			165	87 (58–124)	159	70 (47–91)	0.9

Data are median (IQR) derived from raw data. Data were log-transformed before analysis. Data were analyzed using linear mixed effects models.

^*^ The *p*-value tests time by supplement interaction.

BMIC, breast milk iodine concentration.

At baseline, maternal median Tg concentration was 22 μg/L (12–39), and 22% (81 of 366) of women had elevated Tg (>43.5 μg/L). Maternal MMN supplementation during pregnancy significantly decreased maternal Tg concentration compared with FeFol (*p* < 0.001; Table 2), and significantly decreased the prevalence of elevated Tg (*p* < 0.001; Table 2). Only 3.3% (9 of 271) of the pregnant women tested positive for TgAb at baseline, and with no difference between the supplement groups (*p* = 0.3; Table 2).

There were no differences in the mean or median concentrations between supplement groups in any of the other maternal thyroid function tests (TSH, TT3, TT4, and TT3/TT4 ratio) during pregnancy (Table 2). At baseline, 1.0% (3 of 298) of the mothers had subclinical hypothyroidism and none (0 of 298) had overt hypothyroidism.

At baseline, 2.7% (8 of 298) had overt hyperthyroidism, 2.0% (6 of 298) were hypothyroxinemic, and 10.7% (32 of 298) were affected by subclinical hyperthyroidism, but the prevalence was reduced in both groups at 30 weeks' gestation with no significant overall difference between groups (Table 2).

Maternal MMN supplementation during pregnancy had an effect on cord blood Tg (*p* < 0.001), with lower cord blood Tg concentration in the MMN group. The median cord blood Tg concentration was 100 μg/L (51–140) in the MMN group (*n* = 121) and 127 μg/L (81–191) in the FeFol group (*n* = 108). Furthermore, maternal Tg at 30 week's gestation was associated with cord blood Tg (β coefficient = 0.295 [confidence interval 0.077–0.512], *p* = 0.008).

Maternal MMN supplementation during pregnancy did not have an effect on infant serum Tg concentration postpartum (*p* = 0.9; Table 3). Infant Tg concentrations significantly decreased between 12 and 24 weeks postpartum for both maternal supplement groups (*p* < 0.001).

Median infant TSH concentration in cord blood did not differ between the two supplement groups: 5 mIU/L (4–9) in the MMN group (*n* = 114) versus 6 mIU/L (4–10) in the FeFol group (*n* = 105, *p* = 0.2).

A subanalysis was performed investigating the relationship between gestational age at baseline and maternal Tg, TSH, and TT4 at 30 weeks' gestation in the MMN group; however, no associations were found (data not shown).

## Discussion

Our study shows that supplementing moderately iodine-deficient pregnant women with an MMN supplement containing 300 μg/day of iodine versus FeFol improved maternal iodine status and reduced maternal Tg concentration at 30 weeks' gestation, but had negligible impact on maternal thyroid hormone production. Our results further show that prenatal iodine supplementation alone is not sufficient to ensure adequate iodine status in mothers and infants after delivery.

The estimated maternal iodine intake at baseline was 71 μg/day, which falls far below the recommended intake of 250 μg/day (9). Compliance with supplementation regimen was high and although the median UIC increased at 30 weeks' gestation in the group receiving MMN, the median UIC concentration remained below the recommended threshold of 150 μg/L (9). This threshold is, however, based on UIC from spot urine, which has been shown to have a higher UIC than the 24-hour urine samples (29). The finding is in agreement with earlier studies (5,30) and is perhaps not surprising, considering this study was conducted in rural Gambia where pregnant women are at risk of iodine deficiency (11). The women likely had largely depleted thyroid iodine stores when entering pregnancy, and most of the ingested iodine was taken up by the thyroid for both production of thyroid hormone and rebuilding stores, resulting in a lower fraction excreted in the urine.

The median maternal Tg concentration at baseline (22 μg/L) was elevated above the assay-specific target median of 10 μg/L typically observed in a iodine sufficient population (25). The turnover and excretion of Tg from the thyroid are increased during iodine deficiency as thyroid activity increases to adapt to low iodine intakes (31). The elevated Tg concentration thus suggest thyroid stress, that is increased thyroid activity to produce adequate thyroid hormone in the face of limited iodine supply. The improvement in iodine status in the MMN group decreased the Tg concentration and reduced thyroid stress. Tg is a sensitive biomarker of iodine status throughout the life cycle (25,32). Our results agree well with previous studies on maternal Tg concentration of prenatal iodine supplementation in mild and moderate iodine deficiency (5,7), and findings confirm the sensitivity of Tg to assess changes in thyroid stress in response to changes in iodine intake during pregnancy (33). In mild-to-moderate iodine deficiency, increased thyroid activity can compensate for low iodine intake and maintain euthyroidism in most individuals (34). This is confirmed in our study by TSH, TT3, and TT4 concentrations within the normal reference ranges (26) and low prevalence of maternal hypothyroidism and hypothyroxinemia: the prevalence of subclinical hyperthyroidism was 10.7% in the mothers at baseline, but the prevalence of overt hyperthyroidism was lower at 2.7%. We observed no effect of prenatal iodine supplementation on maternal thyroid function.

Our findings on maternal iodine and thyroid status agree with earlier intervention studies conducted in mild-to-moderately iodine-deficient populations (5,30) and a recent randomized controlled trial of prenatal iodine supplementation (200 μg/day) in mildly iodine-deficient pregnant women (7). The latter study reported improved maternal iodine status and reduced thyroid stress, but no effect on maternal thyroid function, and no long-term benefits on development were observed in children at 5–6 years. It is uncertain how low the iodine intake can be without affecting circulating thyroxine and triiodothyronine concentrations. Our data suggest that thyroid adaptation maintains euthyroidism also at moderately deficient iodine status. Therefore, the lack of effect of iodine supplementation on thyroid hormone concentrations is not surprising. Iodine supplementation of pregnant women is recommended in populations with mild-to-moderate maternal iodine deficiency, particularly where the coverage of iodized salt is low (12), as in our study population (11). The supplemental dose of 300 μg iodine slightly exceeds the recommended dietary intake of 250 μg, but is appropriate and safe considering the degree of iodine deficiency in our population. A high dose of iodine given to chronically iodine-deficient adults may transiently induce hyperthyroidism (35,36), but this was not observed in our study. The 8 cases of overt hyperthyroidism observed at baseline resolved over the course of the study and no cases were observed at 30 weeks' gestation. Furthermore, we observed no decrease in TT4 in supplemented women, as previously reported in a cross-sectional study conducted in a moderately iodine-deficient population (37).

BMIC is strongly associated with the iodine intake of the mother (38), and is the most accurate biomarker of iodine status during lactation (39). BMIC did not differ according to supplement group over the course of the study and the median concentration was more than three times lower than reported in lactating women in iodine replete populations (39). Furthermore, the median UIC in the women at 12 weeks postpartum did not differ between the groups and was below the WHO threshold of 100 μg/L (9). Our data suggest that in a population with persistently low dietary iodine intakes postpartum, prenatal supplemental iodine has minimal long-term effect on excretion in breast milk. In areas of iodine deficiency, maternal postnatal iodine supplementation may be justified to ensure adequate maternal iodine status during lactation, to maintain adequate BMIC and infant iodine status (12).

The estimated iodine intake in the breastfeeding infants in our study was 42 μg/day [assuming a breast milk intake of 0 · 78 L (40)], only half of the dietary iodine requirements (41,42). The elevated Tg concentration observed in the infants from both supplement groups at 12 and 24 weeks postpartum suggests deficient iodine intakes, although no reference range has been established for this age group for the assay used. Circulating Tg levels are typically high in early infancy but fall over the first year of life, likely stabilizing by about 6 months to 2 years of age (43), and the Tg concentrations in the infants in our study followed this pattern. We observed no group differences in the infant Tg concentrations, and thus no long-term effect of maternal prenatal iodine supplementation in the infants. At birth, the cord blood Tg concentration was lower in the MMN group than in the FeFol group, at a ratio comparable with the maternal serum Tg concentration at 30 weeks' gestation. The TSH concentration in cord blood was comparable between the two supplement groups. These findings add to previous observational data and controlled studies reporting associations between Tg, TSH, and thyroid hormone concentrations measured in cord blood and maternal thyroid function (5,7,44,45).

Our finding would support the need for iodine supplementation or the inclusion of iodine in MMN supplements in moderately iodine-deficient populations to improve the iodine intake during pregnancy. However, universal salt iodization is the primary intervention strategy to prevent iodine deficiency in the general population (9). Recent data demonstrate that adequately iodized salt at high coverage meets the requirements of all population groups, including pregnant and lactating women (46). The developing fetal brain is especially vulnerable during the first trimester when the fetus relies on maternal thyroid hormone supply (3). Universal salt iodization ensures adequate iodine intake and sufficient maternal iodine stores when the mother enters pregnancy to maintain optimal fetal thyroid hormone supply at a critical time window when targeted supplementation unlikely is introduced yet. The current WHO position recommending targeted iodine supplementation to pregnant and lactating women primarily in populations with poor coverage of iodized salt remains valid. For this rural Gambian population, important prevention strategies are to ensure that locally produced salt is iodized adequately or that MMN supplementation during pregnancy is standard care rather than FeFol supplementation (47).

The strengths of this study are the randomized design of the ENID trial conducted in a moderately deficient population of pregnant and lactating women, with multiple pre- and postnatal measures of iodine status and thyroid function parameters, along with BMIC during the first six months of lactation. Few well-powered studies have been conducted in areas with moderate iodine deficiency, and even fewer have studied pregnancy, lactation, and infancy and measured the range of biomarkers as done in this study. The drop-out rate was overall low, with only 10% dropouts between birth and 1 year follow-up; furthermore, attrition was low and balanced between study arms. Furthermore, ICP-MS was used to measure UIC and BMIC, the gold standard method for these markers (39,48). Moreover, a 24-hour UIC sample was used rather than a spot UIC and we estimated the iodine intake using the daily iodine excretion obtained from the urine volume measured in the 24-hour urine collection. A limitation of this study is that the intervention of focus was a MMN, and not a trial of an iodine supplement in isolation. Furthermore, we did not measure selenium status in these women. We recognize that the results obtained could be influenced by known or unknown interactions between micronutrients. However, the potential interaction of iron deficiency and folate status was accounted for as the same FeFol dose was used in the two groups and thereby accounted for a possible confounder in this iron-deficient population (49). Infant thyroid hormones were not investigated longitudinally, and infant UIC was not measured, as infant urine was not collected as a part of ENID. This could have improved the interpretation of infant iodine status in this population. Lastly, there were no data available regarding serum TPO levels, thyroid-, or antithyroid medication use in this study population.

In conclusion, we observed that in this moderately iodine-deficient population, supplementation during pregnancy with an iodine-containing MMN improved maternal iodine status. Despite markedly inadequate iodine intake, pregnant women were overall euthyroid and supplemental iodine had limited impact on maternal thyroid hormone production. Our data suggest that prenatal iodine supplementation does not ensure optimal postnatal maternal iodine status, BMIC, and infant iodine status during the first six months after birth. Universal salt iodization should remain the main strategy to prevent iodine deficiency during pregnancy, lactation, and early infancy. If the coverage of iodized salt is poor and prenatal supplementation is required, maternal iodine supplementation should be continued through lactation to increase maternal iodine status, BMIC, and infant iodine status.

## Supplementary Material

Supplemental data
