# Supplementary material for: Effects of an Iodine-Containing Prenatal Multiple Micronutrient on Maternal and Infant Iodine Status and Thyroid Function: A Randomized Trial in The Gambia
Source: Thyroid. 2020 Sep 8;30(9):1355–65. doi: 10.1089/thy.2019.0789 (PMC7482118; doi:10.1089/thy.2019.0789)
Supplement: Supplemental data [file Supp_Data.pdf]

Supplementary Data

Sample Analysis

Urinary iodine concentration (UIC) was measured using inductively coupled plasma mass spectrometry (ICP-MS) (S1), at the Human Nutrition Laboratory of Eidgenössische Technische Hochschule (ETH) Zurich (Zurich, Switzerland). The interassay variability (mean ± standard deviation [SD] [coefficient of variation or CV) of the standard reference materials (ClinChek Urine Control Lot 432 levels 1 and 2) was 123 ± 3.8 μg/L (n = 65) (3.1%) and 504 ± 15.5 μg/L (n = 65) (3.1%). The certified acceptable ranges for levels 1 and 2 are 90–150 and 373–622 μg/L, respectively. World Health Organization criteria based on the median UIC were used to classify adequate iodine intake for pregnant women (≥150 μg/L) (S2). The daily iodine excretion was estimated using the UIC and measured urine volume of the 24-hour urine collection. We estimated the daily iodine intake by assuming that 90% of ingested iodine is excreted in the urine (S3).

Breast milk iodine concentration was measured by ICP-MS at the Medical Research Council Elsie Widdowson Laboratory (Cambridge, UK). Breast milk samples were first diluted (1:50) with a solution of ultragrade tetramethylammonium hydroxide (TMAH) containing tellurium as internal standard (0.5% TMAH, 20 μg/L tellurium). The samples were then analyzed by ICP-MS along with external matrix-matched calibration standards (commercially sourced pooled breast milk; Sera Laboratories International Ltd.). Serum and whole blood (RECIPE Chemicals+, Instruments GmbH and Sero AS) were used as quality controls. The interassay variability (mean ± SD [CV]) of serum level 1 was 50 ± 2.53 μg/L (n = 78) (5.1%) and that of level 2 was 109 ± 3.79 μg/L (n = 78) (3.4%) and that of whole blood was 102 ± 3.15 μg/L (n = 76) (3.0%). The certified acceptable ranges for serum levels 1 and 2 are 36–55 and 79–118 μg/L, respectively, and 95–115 μg/L for whole blood.

Thyroglobulin (Tg) was measured in maternal and infant serum using a sandwich serum-Tg enzyme-linked immunosorbent assay (ELISA) (S4), at the Human Nutrition Laboratory of ETH Zurich. Liquicheck™ Tumor Marker Control (Bio-Rad Laboratories AG, Cressier, Switzerland; LOT. 19990 and LOT. 19970) was used as the standard. Two in-house serum samples (ID1 and ID3) and two in-house serum samples (ID1 and ID2) were used for quality controls of the maternal and infant samples, respectively. For the maternal samples, the interassay variability (mean ± SD [CV]) for ID1 was 21.3 ± 3.2 μg/L (n = 24) (15.0%), and that for ID3 was 28.9 ± 3.4 μg/L (n = 25) (11.7%). For the infant samples, the interassay variability for ID1 was 19.6 ± 3.5 μg/L (n = 24) (17.9%), and that for ID2 was 37.0 ± 4.5 μg/L (n = 21) (12.1%). Elevated Tg concentrations during pregnancy, indicating iodine deficiency, is defined as Tg >43.5 μg/L (S4,S5).

Serum thyrotropin (TSH), total triiodothyronine (TT3), total thyroxine (TT4), and cord blood TSH were measured by immunoassay (IMMULITE; Siemens Healthcare Diagnostics, UK) at the Human Nutrition Laboratory of ETH Zurich using analyte-specific kits and controls. For TSH during pregnancy, we used trimester-specific reference ranges: 0.1–2.5 mIU/L for the first trimester, 0.2–3.0 mIU/L for the second trimester, and 0.3–3.0 mIU/L for the third trimester (S6). For TT4 until gestational week 6, we used the reference range of 58–161 nmol/L; from week 7, we increased the upper reference range by 5% per week until week 15; from week 16 until delivery, we multiplied the non-pregnancy reference range by 1.5 and used the resulting range of 87.0–241.5 nmol/L as a reference (S6). For TT3, we used the manufacturer's reference ranges of 1.3–2.6 nmol/L. The interassay variability (mean ± SD [CV]) for TSH ID1 was 0.43 ± 0.02 μg/L (n = 9) (3.89%), that for ID2 was 5.66 ± 0.29 μg/L (n = 9) (5.17%), and that for ID3 was 31.9 ± 1.63 μg/L (n = 9) (5.1%). The interassay variability for TT3 ID1 was 71.19 ± 10.01 μg/L (n = 7) (14.07%), that for ID2 was 182.9 ± 19.45 μg/L (n = 7) (10.63%), and that for ID3 was 334.14 ± 38.65 μg/L (n = 7) (11.57%). For TT4 ID1, the interassay variability was 4.56 ± 1.11 μg/L (n = 7) (24.39%), ID2 was 11.79 ± 1.14 μg/L (n = 7) (9.67%), and that for ID3 was 14.81 ± 1.57 μg/L (n = 7) (10.6%).

Subclinical hypothyroidism was defined as a high TSH and a normal TT4, overt hypothyroidism was defined as a high TSH and a low TT4, overt hyperthyroidism was defined as a low TSH and a high TT4, subclinical hyper was defined as low TSH and normal TT4, and isolated hypothyroxinemia was defined as a normal TSH and a low TT4.

Maternal thyroglobulin antibody (TgAb) concentrations were analyzed in baseline samples using a serum ELISA (TgAb ELISA, version 2; RSR, Cardiff, UK). The interassay variability (mean ± SD [CV]) for ID1 and ID3 was 150 ± 50 U/mL (n = 7) (4.2%) and 520 ± 150 U/mL (n = 7) (6.6%), respectively. The manufacturer cutoff for TgAb positivity is ≥65 U/mL.

Supplementary Table S1. Nutritional Composition of Daily Intake of Pregnancy Supplements

	MMN	*FeFol*
Iron (mg)	60	60
Folate (μg)	400	400
Vitamin A (RE μg)	1600	
Vitamin D (IU)	400	
Vitamin E (mg)	20	
Vitamin C (mg)	140	
Vitamin B_1 (mg)	2.8	
Vitamin B_2 (mg)	2.8	
Niacin (mg)	36	
Vitamin B6 (mg)	2.8	
Vitamin B_{12} (μg)	5.2	
Zinc (mg)	30	
Copper (mg)	4	
Selenium (μg)	130	
Iodine (μg)	300	

Adapted from Moore et al. (S7).

FeFol, iron and folic acid; MMN, multiple micronutrient; RE, retinol equivalent.

References

S1. Caldwell KL, Maxwell CB, Makhmudov A, Pino S, Braverman LE, Jones RL, Hollowell JG 2003 Use of inductively coupled plasma mass spectrometry to measure urinary iodine in NHANES 2000: comparison with previous method. Clin Chem **49(6 Pt. 1):**1019–1021.

S2. World Health Organization (WHO), United Nations Children's Fund, International Council for Control of IDD 2007 Assessment of Iodine Deficiency Disorders and Monitoring Their Elimination: A guide for Pogramme Managers. Third edition. WHO, Geneva, Switzerland.

S3. Zimmermann MB, Andersson M 2012 Assessment of iodine nutrition in populations: past, present, and future. Nutr Rev **70:**553–570.

S4. Stinca S, Andersson M, Erhardt J, Zimmermann MB 2016 Development and validation of a new low-cost enzyme-linked immunoassay for serum and dried blood spot thyroglobulin. Thyroid **25:**1297–1305.

S5. Stinca S, Andersson M, Weibel S, Herter-Aeberli I, Fingerhut R, Gowachirapant S, Hess SY, Jaiswal N, Jukic T, Kusic Z, Mabapa NS, Nepal AK, San Luis TO, Zhen JQ, Zimmermann MB 2017 Dried blood spot thyroglobulin as a biomarker of iodine status in pregnant women. J Clin Endocrinol Metab **102:**23–32.

S6. Alexander EK, Pearce EN, Brent GA, Brown RS, Chen H, Dosiou C, Grobman WA, Laurberg P, Lazarus JH, Mandel SJ, Peeters RP, Sullivan S 2017 2017 Guidelines of the American Thyroid Association for the diagnosis and management of thyroid disease during pregnancy and the postpartum. Thyroid **27:**315–389.

S7. Moore S, Fulford A, Darboe M, Jobarteh M, Jarjou L, Prentice A 2012 A randomized trial to investigate the effects of pre-natal and infant nutritional supplementation on infant immune development in rural Gambia: the ENID trial: early nutrition and immune development. BMC Pregnancy Childbirth **12:**107.
